# Individualized therapeutic approaches for relapsed and refractory pediatric ependymomas: a single institution experience

**DOI:** 10.1007/s11060-025-05004-1

**Published:** 2025-04-16

**Authors:** Pavel Tinka, Petra Pokorná, Michal Kýr, Zdeněk Pavelka, Klára Vejmělková, Hana Pálová, Jakub Neradil, Marta Ježová, Ondřej Slabý, Jaroslav Štěrba

**Affiliations:** 1 Department of Pediatric Oncology, University Hospital Brno and Faculty of Medicine, Masaryk University, Brno, 62500 Czech Republic; 2 Department of Biology, Faculty of Medicine and Central, European Institute of Technology, Masaryk University, Brno, 62500 Czech Republic; 3 Center for Precision Medicine, University Hospital Brno, Brno, 62500 Czech Republic; 4 Laboratory of Tumor Biology, Department of Experimental Biology, Faculty of Science, Masaryk University, Brno, 62500 Czech Republic; 5 International Clinical Research Center, St. Anne’s University Hospital, Brno, 60200 Czech Republic; 6 Department of Pathology, University Hospital Brno and Faculty of Medicine, Masaryk University, Brno, 62500 Czech Republic

**Keywords:** Refractory, Relapsed, Pediatric ependymoma, Individualized treatment, Targeted therapy, Molecular profiling

## Abstract

**Purpose:**

This retrospective study aims to show a real-life single-center experience with clinical management of relapsed pediatric ependymomas using results from comprehensive molecular profiling.

**Methods:**

Eight relapsed ependymomas were tested by whole exome sequencing, RNA sequencing, phosphoproteomic arrays, array comparative genome hybridization, and immunohistochemistry staining for PD-L1 expression and treated with an individualized approach implementing targeted inhibitors, immunotherapy, antiangiogenic metronomic treatment, or other agents. Treatment efficacy was evaluated using progression-free survival (PFS), overall survival (OS), survival after relapse (SAR), and PFS ratios.

**Results:**

Genomic analyses did not reveal any therapeutically actionable alterations. Surgery remained the cornerstone of patient treatment, supplemented by adjuvant radiotherapy. Empiric agents were chosen quite frequently, often involving drug repurposing. In six patients, prolonged PFS after relapse was seen because of immunotherapy, MEMMAT, or empiric agents and is reflected in the PFS ratio ≥ 1. The 5-year OS was 88%, the 10-year OS was 73%, the 2-year SAR was 88%, and the 5-year SAR was 66%.

**Conclusion:**

We demonstrated the feasibility and good safety profile. Promising was the effect of immunotherapy on ZFTA-positive ependymomas. However, further research is required to establish the most effective approach for achieving sustained remission in these patients.

**Supplementary Information:**

The online version contains supplementary material available at 10.1007/s11060-025-05004-1.

## Introduction

Ependymomas account for about 5% of primary intracranial tumors in children and are the third most common malignant intracranial neoplasm [1]. These tumors can develop in all compartments of the central nervous system (CNS), most frequently in the infratentorial space, followed by supratentorial space, and rarely in the spinal cord [2]. The 2021 WHO classification [3] recognizes several molecular subgroups, primarily based on epigenetic classification published by Pajtler et al. in 2015 [4]. Among these, Posterior Fossa A (PFA) or ZFTA fusion-positive (ZFTA +) subgroups are predominantly observed in infratentorial or supratentorial regions, respectively [4], and confer worse prognosis with a 5-year overall survival of 68% and 75% [4].

Despite initial complete resection and focal radiotherapy, which is considered standard initial management for risky molecular subgroups [5, 6], almost 50% of pediatric ependymomas recur [5, 7]. Re-resection and focal re-irradiation are crucial parts of relapse treatment [8, 9], but their effectiveness is far from ideal [10]. The development of other treatment modalities is necessary.

Ependymomas are generally known to harbor very few somatic alterations [11]. Some genomic rearrangements like CDKN2A deletion in ZFTA + [12] and 1q gain [13] or 6q loss [14] in PFA ependymomas have been described as prognostic markers. However, well-established genomic findings that are found in other CNS tumors and could serve as predictive markers [15] are not characteristic of these tumors and their tumorigenesis is considered to be driven primarily by epigenetic mechanisms, as supported by new findings highlighting overexpression of EZHIP protein leading to H3K27me3 hypomethylation observed in PFA ependymomas [16].

Therefore, targeted therapies in relapsed pediatric ependymoma remain challenging. Valproic acid, repurposed as an epigenetic modifier [17], is currently being tested in the SIOP-EP II trial [18]. Among other targeted approaches, there is limited experience with immune checkpoint inhibitors (ICIs) [19, 20] and their combination with mTOR inhibitors [21], mTOR inhibitors with anti-VEGF antibody bevacizumab [22], and tyrosine kinase inhibitors (TKIs) [23, 24]. While all these approaches have demonstrated safety, their efficacy remains uncertain. Further studies of potent tyrosine kinase inhibitors (TKIs) are currently ongoing [25].

This article presents a case series of eight relapsed or refractory (R/R) ependymoma patients, in which the subsequent treatment strategy implemented various novel treatment modalities, including TKIs, immunotherapy, metronomic therapy, or various other agents showing preclinical evidence of antitumor activity.

## Methods

### Study design

Patients were identified using an institutional database and included in this retrospective study if they were treated for R/R ependymoma based on the recommendation from the Molecular Tumor Board Committee (MOTB) from January 2016 to December 2023. Data collected from the electronic medical record included patient demographics, histopathological features, molecular profile, standard treatment, individualized treatment, toxicity related to individualized treatment, duration of therapy, and radiographic response. The study has been approved by the Masaryk University Ethical Committee and the Ethics Committee of University Hospital Brno.

### Molecular analyses

All patients underwent comprehensive molecular profiling of tumor tissue, which included whole exome sequencing (WES), RNA sequencing (RNAseq), phosphoproteomic arrays, array comparative genomic hybridization (aCGH), and immunohistochemistry (IHC) staining for PD-L1 expression. Molecular results were interpreted by multidisciplinary MOTB, consisting of several molecular biologists, pathologists, clinical pharmacologists, pharmacists, data managers, and neuro-oncologists, with regular participation of several international experts, who collectively discussed each case and decided on the next treatment steps reflecting molecular results, with their levels of evidence according to OncoKB database [26] where the highest Level I represents clinical trial evidence, Level II represents clinical evidence (case reports or series), Level III represents experimental evidence (in vivo, in vitro) and Level IV is reserved for no clinical or experimental evidence. Because of the small number of pediatric ependymoma patients and the general lack of pediatric trials, we approximate the evidence to the trials and experiences from the adults. Transcriptomic and phosphoproteomics data in our study were considered a low level of evidence (Level IV). Another important factor represents the availability and toxicity of potential drugs using a staggered approach to avoid severe side effects.

### Toxicity assessment

Adverse events (AEs) were continuously evaluated and graded according to Common Terminology Criteria for Adverse Events (CTCAE) v5.0. Toxicity grades 3 and higher led to dose modification or treatment discontinuation.

### Treatment assessment

MRI was used to assess response using Response Assessment in Neuro-Oncology (RANO) criteria. Complete response (CR) is defined by the absence of disease in MRI scan and clinically stable patient, reducing or no steroids, partial response (PR) by the 50% decrease of measurable and no progression of non-measurable disease in MRI scan and clinically stable patient, reducing or no steroids, progression disease (PD) by the 25% or more increase or any new lesion in MRI scan and/or clinical deterioration of the patient, stable disease (SD) not fulfilling criteria for CR, PR or PD and clinically stable patient. Treatment efficacy was evaluated using progression-free survival (PFS): time from the last event (diagnosis, progression) to the new event (progression, death, or last follow-up visit), overall survival (OS): time from the diagnosis to the last follow-up visit or death, survival after relapse (SAR): time from the diagnosis to the first progression, and PFS ratio: ratio between the last PFS and the previous one.

### Statistical analysis

R software has been used to calculate the Kaplan–Meier survival analysis of OS, PFS, and PFS ratio.

## Results

The presented cohort is comprised of tumors with primary locations in the posterior fossa and supratentorial space in six and two cases, respectively. Accordingly, the molecular subtypes were represented by six PFA and two ZFTA + tumors. The median age at diagnosis was five years. Initially, all patients underwent maximal safe resection, and 5 of them followed by adjuvant radiotherapy. In the remaining cases, radiotherapy was deferred due to young age at diagnosis or parental refusal. Five patients received adjuvant chemotherapy per SIOP-EP II stratum 3 [18] and ACNS0831 [27] protocols. The best response during the first progression-free survival was CR in six cases and PR in two cases. Subsequent relapses ranged from one to five and were mainly localized, with one metastatic relapse into the spinal cord and cerebrospinal fluid (CSF). Additional clinical and histopathological characteristics of all patients are summarized in Table 1.

**Table 1:** Overview of clinicopathological characteristics of the cohort, initial treatment, and subsequent number of relapses in the cohort

	Patient no							
	1	2	3	4	5	6	7	8
Sex	F	F	M	M	M	F	M	F
Age (years) at diagnosis	1.04	1.63	1.16	8.25	15.01	9.71	5.44	4.49
Histopathological grade	III	III	III	III	III	II	III	II
Molecular subtype	PFA	PFA	PFA	ZFTA	PFA	ZFTA	PFA	PFA
Location	PF	PF	PF	ST	PF	ST	PF	PF
Metastatic at presentation	No	No	No	No	No	No	No	No
Radicality of surgical resection	STR	GTR	GTR	GTR	STR	GTR	STR	STR
Initial radiotherapy (brain focal)	NA	45 Gy	45 Gy	59,4 Gy	NA	NA	45 Gy	54 Gy
Type of radiotherapy	NA	proton	photon	photon	NA	NA	photon	photon
Initial chemotherapy	SIOP-EP-II	ACNS0831	None	SIOP-EP-II	None	None	ACNS0831	None
The best response in PFS1	CR	CR	CR	CR	PR	CR	CR	PR
Total no. of relapses	5	4	2	1	5	1	1	1
Metastatic relapses	0	0	0	0	0	0	1	0

*F* female, *M* male, *PFA* posterior fossa A, *ZFTA* ZFTA-fusion positive, *PF* posterior fossa, *ST* supratentorial location, *STR* subtotal resection, *GTR* gross total resection, *CR* complete response, *PR* partial response

Genomic analyses did not reveal any established therapeutically actionable alterations, such as activating oncogene variants or inactivating tumor suppressor variants. However, phosphoproteomic arrays indicated significantly increased phosphorylation of different receptor kinases in three patients. Additionally, three patients were eligible for anti-PD-1 immunotherapy based on the results of IHC staining for PD-L1 protein expression. Consequently, the administration of tyrosine kinase inhibitors (TKIs) or anti-PD-1 represented the only biomarker-driven strategy utilized within the cohort. Surgery remained the cornerstone of patient treatment, supplemented by adjuvant radiotherapy, CHT protocols as well as experimental approaches, including the aforementioned biomarker-based therapeutics, dendritic cell (DC) vaccine, the MEMMAT protocol [28], and so-called empiric agents chosen based upon preclinical studies showing possible anticancer effects, often involving drug repurposing [17].

**Fig. 1:**
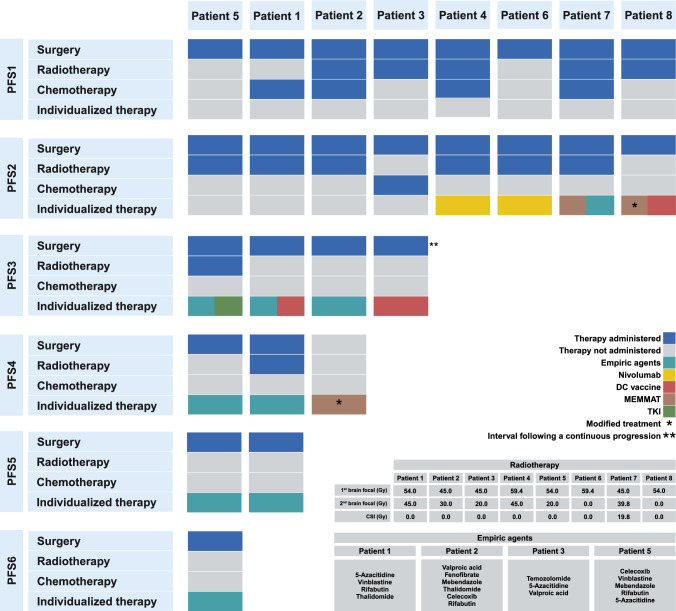
Graphical summary of treatment modalities administered during the course of the disease. Six patients were administered immunotherapy, which consisted of either nivolumab or a DC vaccine. In two of these cases (patients 4 and 6), immunotherapy was the sole experimental approach used during the disease course on top of standard treatment options and led the patients to sustained CR. In patient 3, the DC vaccine sustained CR after a period of continuous progression on empiric treatment with nivolumab (not shown in this figure). In one case, immunotherapy was not sufficient to sustain CR and upon recurrence, was replaced by empiric treatment (patient 1). In patient 8, immunotherapy was administered alongside empiric agents or combined with the MEMMAT protocol, respectively. Additionally, one patient underwent all available experimental approaches throughout the prolonged disease course (patient 2), immunotherapy and targeted treatment were used without effect at the end of the disease course and are not shown in this figure. In the remaining two cases, one patient was treated with MEMMAT and empiric agents (patient 7) and one with empiric treatment, used alone or in combination with TKIs (patient 5). Created in BioRender. https://BioRender.com/n52p573

**Fig. 2:**
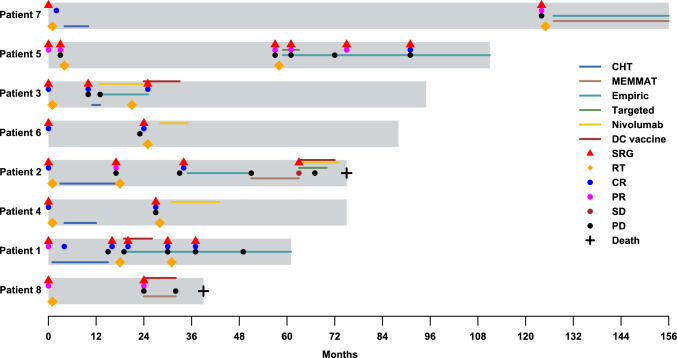
Swimmer plot depicting scheduling of the respective treatment options and response indicators in the presented cohort. Nearly every disease progression was followed by surgical intervention, though achieving complete resection was difficult in patients 5 and 8. For patient 5, only the last surgery was successful and led to the first CR. In patient 7 the tumor is currently responding to the MEMMAT protocol by decreasing its size on the MRI scan. Patients 3, 4, and 6, who received immunotherapy as maintenance following CR, have sustained CR for at least 3 years from the last relapse. Despite maximal treatment efforts, patients 1, 2, and 5 repeatedly relapsed, although empiric treatment led to the prolongation of PFS in patient 5. Patients 2 and 8 died within 6 years of diagnosis. In patient 2, the tumor progressed despite immunotherapy, TKI, empiric treatment, and MEMMAT protocol. Patient 8 didn’t respond to the MEMMAT protocol. However, its intrathecal component couldn’t be used. Patient 1 didn’t significantly respond to any individualized modality except the DC vaccine and is slowly progressing up to the endpoint of this study

The group of empiric agents comprised several agents with suggested benefits in epigenetically-driven tumors, such as 5-azacitidine (5-AZA) [29], valproic acid [30], rifabutin or mebendazole [31, 32], as well as some agents implemented within MEMMAT administered outside of the respective trial. When feasible, biomarker-driven therapy options were preferred, with the remaining experimental approaches being chosen according to the availability of the respective modalities and patient eligibility. The treatment summary is detailed in Fig. 1, and the treatment schedule is visualized in Fig. 2. Individual case descriptions are available within the Supplementary data file.

**Fig. 3:**
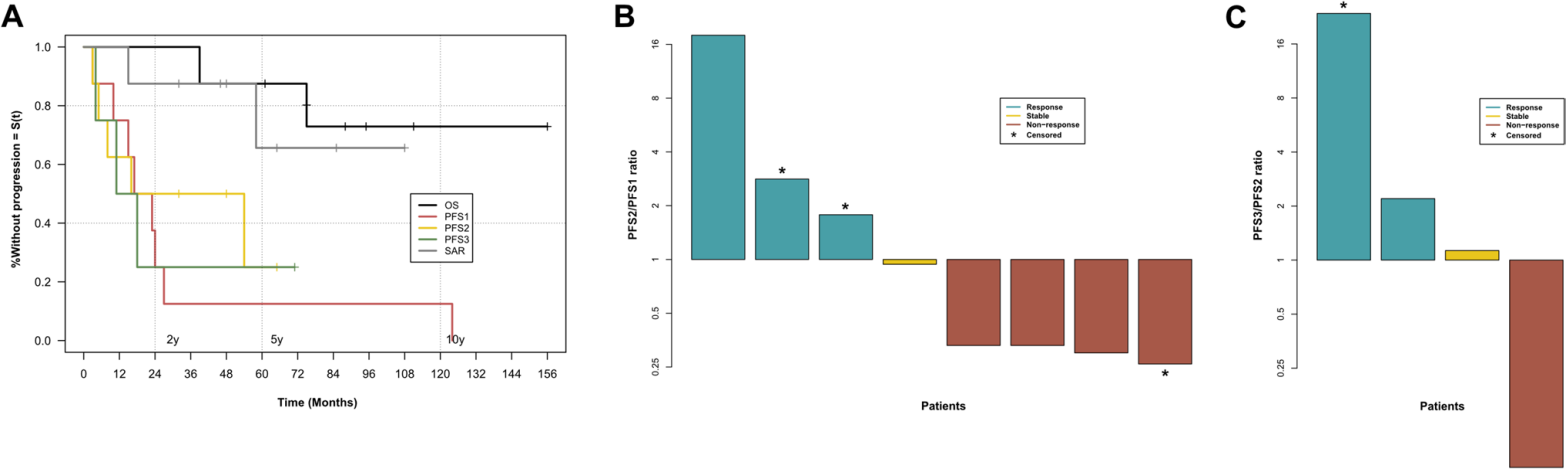
Survival analysis of the cohort, including **A** Kaplan–Meier plots of OS, PFS1, PFS2, PFS3, and SAR, **B** Waterfall plot of PFS2/PFS1 ratio, and **C** Waterfall plot of PFS3/PFS2 ratio. For better visualization, Y-axes in Figures B and C are on a log2 scale. In Figure A, we can see the late relapse of patient 7 (more than 10 years from the initial diagnosis), underlying the necessity for long-term follow-up. Figure B shows the effective ratio (≥ 0.8) in four patients with one censored case whose long PFS1 places them below one but could be changed within the longer observation. Figure C displays the PFS3/PFS2 ratio, where three of four patients exceeded the 0.8 cut-off

The median OS for the cohort was 81.5 months (range 39–156 months), with 2-year, 5-year, and 10-year OS rates of 100%, 88%, and 73%, respectively (see Fig. 3A). Two patients died due to disease progression, with OS of 75 and 39 months. The median SAR was 48 months (range 15–108 months). 2-year SAR was 88%, whereas 5-year SAR was 66%.The median PFS was 20 months for PFS1 (range 3–124, n = 8), 24 months for PFS2 (range 3–65, n = 8), 14.5 months for PFS3 (range 4–71, n = 4), 9 months for PFS4 (range 7–12, n = 3) and 15.5 months for PFS5 (range 12–19, n = 2). One patient has a PFS6 lasting 22 months without an event at the study’s endpoint. To assess treatment efficacy in this small group, we applied a PFS ratio developed by Von Hof [33] using a ≥ 0.8 cut-off for disease control and ≥ 1.2 for a response. Four patients met at least disease control for PFS2/PFS1, two patients for PFS3/PFS2, one for PFS4/PFS3, two for PFS5/PFS4, and one for PFS6/PFS5 (see Fig. 3B-E and Supplementary Table 1).

The toxicity profile of the individualized treatment was generally favorable and is detailed in Table 2. Grade 4 AEs occurred twice, involving intracranial bleeding or atypical pneumonitis related to 5-AZA or nivolumab, respectively. Grade 3 AEs occurred four times, including cytopenia from empiric treatment and neurocognitive impairment due to the MEMMAT protocol. Grade 1 or 2 AE appeared frequently, mainly characterized by cytopenia or elevated liver enzymes. All AEs were appropriately managed, and there were no treatment-related deaths.

**Table 2:** Adverse events encountered while administering individualized treatment

Patient	AE	Grade (CTCAE v. 5.0)	Responsible agent
Patient 1	Cytopenia	3	VBL
	ALT/AST elevation	2	n.d
	Nausea/Vomiting	1	n.d
Patient 2	Nephropathy	2	Celecoxib
	Atypical pneumonia	4	Nivolumab
	ALT/AST elevation	2	n.d
	Anorexia	1	n.d
	Cytopenia	3	TMZ
Patient 3	Skin toxicity	2	Nivolumab
	Nausea/Vomiting	1	n.d
Patient 4	ALT/AST elevation	2	Nivolumab
Patient 5	Cytopenia	3	VBL, 5-AZA
	Intracranial bleeding	4	5-AZA
	Skin toxicity	2	Cetuximab
	Nefropathy	2	Celecoxib
	ALT/AST elevation	1	n.d
Patient 6	ALT/AST elevation	2	Nivolumab
	Skin toxicity	1	Nivolumab
Patient 7	Cytopenia	1	MEMMAT
	Neurocognitive impairment	1	MEMMAT
	ALT/AST elevation	1	n.d
Patient 8	Cytopenia	1	mod. MEMMAT
	Anorexia	1	mod. MEMMAT

*5-AZA* 5-azacytidine, *n.d.* responsible agent could not be determined, *TMZ* temozolomide, *VBL* vinblastine

## Discussion

Current treatment strategies after relapse yield unsatisfactory outcomes. Re-resection and focal re-irradiation have proven effective [8, 34], which is also reflected in our cohort, where the best response in the second PFS was achieved by re-resection in every case and was followed by radiotherapy in 7 cases, with 3 of them receiving radiotherapy for the first time. However, both resection and radiation carry risks of severe morbidity and should be carefully considered. While re-irradiation could be performed with acceptable short-term toxicity [35], its feasibility could be limited by short intervals between prior radiation courses or proximity to critical structures in the radiation field. Modifying focal re-irradiation to cerebrospinal irradiation (CSI) in focally relapsed patients has demonstrated greater efficacy [36], but is limited by worse long-term toxicity of CSI [37] and was not used in this cohort.

The role of chemotherapy is currently evaluated in the SIOP-EP II trial [18], where it serves as a frontline therapy in different combinations of radiotherapy and residual disease. Early results of the recent ACNS 0831 trial [27] randomizing patients with and without adjuvant chemotherapy have shown possible effects in some ependymoma patients, but further analyses are needed and proceeding. Historically, the effectiveness of frontline chemotherapy in ependymoma was considered uncertain, even with high-dose chemotherapy regimens [10], in many phase II studies indicating prolonged remission in individual cases but no significant global improvement in OS. In relapsed treatment, adjuvant chemotherapy failed to improve OS in the large cohort of ependymoma patients from German HIT-REZ studies [38]. However, chemotherapy remains an option for relapsed patients who can tolerate it and for whom local control cannot be achieved with radiation or surgery [39]. It may also serve to delay radiotherapy or to reduce tumor size before the second-look surgery [40]. In this cohort, we used chemotherapy mainly in frontline therapy except for patient 3 where we followed the ACNS0831 protocol [27] after resection without significant effect.

Molecularly driven targeted therapies have become a promising option for R/R solid tumors. However, this strategy is limited in ependymomas, as these tumors are generally known to harbor very few alterations and lack genomic findings that could serve as predictive markers [15]. Nonetheless, the role of protein kinase signaling has been implied in ependymoma tumorigenesis [41], prompting several ongoing studies evaluating TKIs targeting RET, ALK, ROS, IGFR, PDGFR, FGFR, or EGFR, as well as inhibitors targeting PI3K, mTOR, KIT, and CDK4 [25]. In our cohort, no targetable variants were found on the DNA level. However, phosphoproteomic analyses enabled the detection of increased phosphorylation of EGFR, PDGFRβ, or Ins-R, in three patients, one of whom received a corresponding TKI without a recognizable benefit. Despite prior evidence supporting receptor tyrosine kinase involvement in ependymoma, phase II studies, as well as our own clinical experience have not proven the efficacy for specifically erlotinib and sunitinib [23, 24].

Other potential therapeutic strategy involves targeting the tumor’s interaction with its microenvironment, such as PD-L1 inhibition (discussed later) or anti-angiogenic therapy with anti-VEGF antibody bevacizumab. While early data on bevacizumab suggested promise [40], subsequent studies in ependymoma didn’t confirm its efficacy [22, 42]. This might be explained by findings demonstrating that the ependymoma vasculature has less angiogenic activity and is generally more mature compared to aggressive high-grade gliomas [25]. In this cohort, bevacizumab was used only in combination with other antiangiogenic agents and intrathecal chemotherapy in MEMMAT protocol which is discussed further.

Immunotherapy is increasingly considered for R/R pediatric brain tumor patients, with current reports largely consisting of pilot studies or phase I trials utilizing immune checkpoint inhibitors (ICIs) focused primarily on establishing safety and dose tolerance while monitoring survival endpoints [19, 43]. Similarly, in our cohort, we considered anti-PD-1 blockade if there was a biomarker-based rationale [44] for its administration. We assume a positive impact of nivolumab in a maintenance setting in two ZFTA + patients, in whom their second CR was achieved through resection and radiotherapy. Notably, the subsequent addition of nivolumab led to a prolongation of their second PFS. PD-L1 expression and the possibility of the anti-PD-1 blockade in the ZFTA + subtype have already been described by some studies [19, 20] and might suggest a viable future direction. On the other hand, nivolumab was unsuccessful in PFA patient 3 despite high PD-L1 positivity. It correlates with findings that the immunosuppressive phenotype of PFA ependymoma is rather associated with wound healing and tissue remodeling [25] than PD-L1 interaction. Furthermore, nivolumab was used during tumor progression in this case, not as a maintenance in CR, suggesting the importance of the proper position of the ICIs in the treatment schedule. Apart from ICIs, DC vaccines have also been utilized in some patients. Data regarding their effect on pediatric ependymomas are limited, but reports of mixed R/R patient cohorts showed promising preliminary results [45, 46]. We have observed significantly increased PFS3/PFS2 ratios in PFA patients 1 and 3, who used the vaccine in CR. Patients 2 and 8 started vaccination with residual disease and progressed without significant prolongation of the interval. The future direction of immunotherapy for pediatric brain tumors represents CAR-T cell therapy. However, only the potential targets of ependymoma cells for chimeric receptors, have been recently discovered [47], and many further studies are needed.

Interestingly, an antiangiogenic and intrathecal MEMMAT regimen, which has already shown a prolongation of long-term survival in recurrent medulloblastoma patients [48] led PFA patient 7 into PR. Although this effect was not reflected in the PFS ratio it is the only instance in which we were able to demonstrate curative potential instead of using it in a maintenance setting after achieving CR by other modalities. In patients 2 and 8, we did not see the benefit of modified MEMMAT despite the presence of bevacizumab, possibly due to the inability to use its intrathecal component, which represents an integral part of this regimen.

Empiric therapeutic options included heterogeneous repurposed agents with potential activity in preclinical models or other oncological diagnoses. The most extensively studied compound is valproic acid, evaluated in stratum III of the SIOP-EP-II trial [18], known for its ability to inhibit histone deacetylase activity [30]. Another potential epigenetic agent, 5-AZA, inhibits DNA methylation and has been tested in several pediatric cancer models, including brain tumors [29]. Its CSF penetration via oral administration remains uncertain; however, an intraventricular delivery was tested by Sandberg et al. [49] and demonstrated safety. Other repurposed modalities lacked ependymoma-specific rationale but were considered based on their CSF penetration and preclinical activity in other CNS tumors. Namely, mebendazole was chosen for its good CSF penetration and preclinical activity in MBL models [31] with possible antiangiogenic effect. Rifabutin (ansamycin) was selected due to its putative targeting of HSP-90 and NF-κB pathways [32], which showed increased activity in transcriptomic data in our cohort. Since these agents were primarily administered as a supportive treatment in combination with other treatment strategies, independent efficacy remains difficult to assess. The only clear effect in the context of a residual tumor was seen in patient 5, as supported by prolonged PFS4/PFS3 and PFS5/PFS4 ratios. However, in patients 1 and 2, the sole administration of empiric agents didn’t yield any benefit. Therefore, such agents might potentially serve as a supportive backbone to other approaches aiming at maximal multimodal disease control, rather than as stand-alone therapies.

Compared to other studies reporting a 5-year SAR of around 40% [8, 50], we achieved a 66% rate. However, this observation might be influenced by the small size of our cohort. From an individual perspective, we attained a prolongation of PFS in selected cases, suggesting a potential benefit of the respective chosen strategies. Based on our experience, surgery, and radiotherapy remain the cornerstone of relapse treatment, but their use should be carefully weighed against the risk of potential severe morbidity. Early molecular testing of tumor samples and their microenvironment should be integrated into initial management to guide subsequent therapeutic decisions. In our cohort, immunotherapy was the most promising approach, particularly as maintenance therapy in CR. ICIs demonstrated efficacy in PD-L1 positive ZFTA + tumors, while in cases with negative microenvironment biomarkers, DC vaccines may serve as an alternative. When immunotherapy is unavailable or significant residuum is present, the MEMMAT protocol might be considered, though it serves only as palliative care aimed at slowing the tumor growth and comes with the risk of neurocognitive impairment and requires frequent outpatient visits. Other modalities, like repurposed valproic acid or 5-AZA, while showing potential, require further evaluation to confirm their anti-cancer effect and determine the optimal scheduling, as we observed discordant responses among our patients. However, it could be used as a support to other modalities or an alternative in case of the unavailability of previous approaches. TKIs and other molecularly targeted agents remain marginal in relapse management of pediatric ependymoma due to the lack of underlying actionable alterations.

In conclusion, identifying a rational therapeutic target based on the current understanding of the molecular background of ependymoma is still unsatisfactory. More studies are needed to elucidate signaling pathways crucial for ependymoma development that could be explored therapeutically. The application of additional strategies presented within this report appears feasible with a favorable safety profile, but their efficacy warrants further investigation.

## Supplementary Information

Below is the link to the electronic supplementary material.

Supplementary file1 (DOCX 21 KB)

## Data Availability

Additional details of the individual patient’s diagnostics, treatment, and follow-up are available from the corresponding author upon reasonable request.
