# Supplementary material for: Individualized therapeutic approaches for relapsed and refractory pediatric ependymomas: a single institution experience
Source: J Neurooncol. 2025 Apr 16;173(2):479–88. doi: 10.1007/s11060-025-05004-1 (PMC12106150; doi:10.1007/s11060-025-05004-1)
Supplement: Supplementary file 1 — Supplementary file1 (DOCX 21 KB) [file 11060_2025_5004_MOESM1_ESM.docx]

**Supplementary data**

**Individual case descriptions**

Patient 1 received weekly DC vaccine and vinblastine (VBL) during PFS3 in the absence of measurable disease, resulting in a PFS3/PFS2 ratio of 2.20. Following another complete resection in PFS4, the patient underwent re-radiotherapy and empiric treatment, continuing VBL weekly alongside 5-azacytidine, rifabutin, and thalidomide for drug repurposing. A subsequent resection in PFS5 was performed, and the patient is currently in palliative care with a slowly progressing tumor and an overall survival (OS) of 61 months.

Patient 2 initiated empiric treatment during PFS3, which included valproic acid, fenofibrate, mebendazole, thalidomide, celecoxib, and rifabutin after complete resection. The PFS3/PFS2 ratio was 1.13. With an unresectable tumor in PFS4, which lasted 12 months, the treatment was switched to the MEMMAT protocol without intrathecal chemotherapy. After another progression, immunotherapy with a DC vaccine and nivolumab was started alongside regorafenib targeting hyperphosphorylated PDGFRβ without effect. The patient passed away with an OS of 75 months.

Patient 3 began nivolumab with Valproic acid, Temozolomide and 5-Azacitidine treatment following disease progression after PFS2 containing complete resection and CHT but continued to progress during this therapy. A subsequent surgery achieved a third complete remission, followed by nearly a year of maintenance therapy with a DC vaccine considered as a PFS3. OS reached 95 months, with PFS3 lasting 71 months compared to 3 months for PFS2, reflecting a PFS3/PFS2 ratio of 23.67.

Patient 4 was treated with nivolumab as maintenance therapy following complete resection and radiotherapy, which was discontinued after one year. The OS is 75 months, with a PFS2 of 48 months versus 27 months for PFS1, resulting in a PFS2/PFS1 ratio of 1.78.

Patient 5 received an individualized treatment during PFS3, including cetuximab to inhibit EGFR and an empiric regimen of weekly VBL, rifabutin, mebendazole, celecoxib, and 5-azacytidine, though significant improvement was not achieved. During PFS4, partial resection and empiric treatment were continued, with a PFS4/PFS3 ratio of 2.25. After another partial resection in PFS5, treatment continued, achieving a PFS5/PFS4 ratio of 2.11. Following further progression, a complete resection was performed, marking the first complete remission (CR). PFS6 is ongoing, currently at 22 months, with adjuvant empiric treatment, and the patient’s OS is 111 months.

Patient 6, similar to patient 4, received nivolumab as maintenance following complete resection and radiotherapy. The therapy was discontinued after one year. The OS is 88 months, with a PFS2 of 65 months versus 23 months for PFS1, giving a PFS2/PFS1 ratio of 2.82.

Patient 7 experienced a metastatic relapse 10 years after initial complete remission. Due to limited molecular targets, the MEMMAT protocol with rifabutin was employed as adjuvant therapy. The patient’s PFS2 is 32 months, achieving partial remission (PR) during treatment, with an OS of 156 months.

Patient 8 had suboptimal local control with an inoperable residual tumor and progressed 24 months after first-line therapy. With no actionable targets, a modified MEMMAT protocol, excluding intrathecal therapy due to a ventriculoperitoneal shunt, was combined with a DC vaccine. The patient progressed after 8 months and passed away with an OS of 39 months.

**Supplementary table 1: Survival intervals.**

|  | **Patient no.** | | | | | | | |
| --- | --- | --- | --- | --- | --- | --- | --- | --- |
|  | **1** | **2** | **3** | **4** | **5** | **6** | **7** | **8** |
| **OS** | 61 | 75 | 95 | 75 | 111 | 88 | 156 | 39 |
| **SAR** | 46 | 58 | 85 | 48 | 108 | 65 | 32 | 15 |
| **PFS1** | 15 | 17 | 10 | 27 | 3 | 23 | 124 | 24 |
| **PFS2** | 5 | 16 | 3 | 48 | 54 | 65 | 32 | 8 |
| **PFS3** | 11 | 18 | 71^*^ | NA | 4 | NA | NA | NA |
| **PFS4** | 7 | 12 | NA | NA | 9 | NA | NA | NA |
| **PFS5** | 12 | NA | NA | NA | 19 | NA | NA | NA |
| **PFS6** | NA | NA | NA | NA | 22 | NA | NA | NA |
| **PFS2/PFS1** | 0,33 | 0,94 | 0,3 | 1,78 | 18 | 2,82 | 0,26 | 0,33 |
| **PFS3/PFS2** | 2,20 | 1,13 | 23,67 | NA | 0,07 | NA | NA | NA |
| **PFS4/PFS3** | 0,64 | 0,67 | NA | NA | 2,25 | NA | NA | NA |
| **PFS5/PFS4** | 1,71 | NA | NA | NA | 2,11 | NA | NA | NA |
| **PFS6/PFS5** | NA | NA | NA | NA | 1,15 | NA | NA | NA |
| **Death** | no | yes | no | no | no | no | no | yes |

* Interval after a period of continuous progression
